# Risk factors for mortality in patients with acute exacerbation of cor pulmonale in plateau

**DOI:** 10.1186/s12890-023-02509-1

**Published:** 2023-07-03

**Authors:** Xiaokai Feng, Chenlu Yang, Zerui Sun, Wanrong Kan, Xiang He, Yongxin Chen, Yajun Tuo

**Affiliations:** 1 Department of Respiratory and Critical Care Medicine, Qinghai Provincial People’s Hospital, 2 Gonghe Road, Xining, 810007 Qinghai Province China; 2 Department of Epidemiology and Biostatistics, School of Basic Medicine, Institute of Basic Medical Sciences Chinese Academy of Medical Sciences, Peking Union Medical College, Beijing, China; 3 Department of Respiratory and Critical Care Medicine, Beijing Institute of Respiratory Medicine and Beijing Chao-Yang Hospital, Capital Medical University, Beijing, China; 4 Department of Geratology, Qinghai Red Cross Hospital, 55 South Street, Xining, 810000 Qinghai Province China

**Keywords:** Cor pulmonale, Mortality, High altitudes, Pulmonary hypertension, Risk factors

## Abstract

**Background:**

The risk factors for mortality might differ between patients with acute exacerbation of chronic pulmonary heart disease in plains and plateaus, while there is a lack of evidence.

**Method:**

Patients diagnosed with cor pulmonale at Qinghai Provincial People’s Hospital were retrospectively included between January 2012 and December 2021. The symptoms, physical and laboratory examination findings, and treatments were collected. Based on the survival within 50 days, we divided the patients into survival and death groups.

**Results:**

After 1:10 matching according to gender, age, and altitude, 673 patients were included in the study, 69 of whom died. The multivariable Cox proportional hazards analysis showed that NYHA class IV (HR = 2.03, 95%CI: 1.21–3.40, P = 0.007), type II respiratory failure (HR = 3.57, 95%CI: 1.60–7.99, P = 0.002), acid-base imbalance (HR = 1.82, 95%CI: 1.06–3.14, P = 0.031), C-reactive protein (HR = 1.04, 95%CI: 1.01–1.08, P = 0.026), and D-dimer (HR = 1.07, 95%CI: 1.01–1.13, P = 0.014) were risk factors for death in patients with cor pulmonale at high altitude. Among patients living below 2500 m, cardiac injury was a risk factor for death (HR = 2.47, 95%CI: 1.28–4.77, *P* = 0.007), while no significant association was observed at ≥ 2500 m (*P* = 0.057). On the contrary, the increase of D-dimer was only a risk factor for the death of patients living 2500 m and above (HR = 1.23, 95% CI: 1.07–1.40, *P* = 0.003).

**Conclusion:**

NYHA class IV, type II respiratory failure, acid-base imbalance, and C- reactive protein may increase the risk of death in patients with cor pulmonale. Altitude modified the association between cardiac injury, D-dimer, and death in patients with cor pulmonale.

**Supplementary Information:**

The online version contains supplementary material available at 10.1186/s12890-023-02509-1.

## Background

Cor pulmonale refers to the enlargement and failure of the right heart due to increased vascular resistance (e.g., chronic long-standing alveolar hypoxia) or elevated pulmonary blood pressure [1]. Chronic cor pulmonale typically results in right ventricular hypertrophy (RVH) [1–3], while acute cor pulmonale usually leads to dilatation [3, 4]. Only diseases originating from the pulmonary circulation system can be classified as cor pulmonale. In the United States, pulmonary heart disease accounts for 10 -30% of all heart failure admissions, and over 40% of chronic lung disease patients show signs of pulmonary heart disease at autopsy [3]. The main causes include vascular changes from tissue damage (e.g., disease and hypoxic injury) or chronic hypoxic pulmonary vasoconstriction [1, 5]. Untreated cor pulmonale increases the risk of death [1, 3, 6, 7].

High-altitude areas (> 2500 m) may be destinations for vacation or work (about 40 million people each year) or permanent residences for many people (about 140 million) [8]. The decrease in atmospheric pressure also reduces the fraction of inhaled oxygen, leading to various exaggerated systemic reactions, such as acute mountain sickness, high-altitude cerebral edema, high-altitude pulmonary edema, and high-altitude pulmonary hypertension [9]. Long-term exposure to high altitude can lead to chronic hypoxia, permanently remodeling the pulmonary blood vessels, leading to high-altitude pulmonary hypertension [8, 9], and possibly developing cor pulmonale. The prevalence of cor pulmonale in males of the Pamir Mountains is 4.6% [10]. But there is a lack of research on risk factors for mortality in patients with cor pulmonale in plateau.Early identification of high-altitude pulmonary hypertension and cor pulmonale is crucial for preventing complications and death. However, the evidence about the risk factors of pulmonary circulation disease at high altitude is very limited.

Based on that most patients with cor pulmonale are over 40 years old [11], the purpose of this study was to explore the risk factors of death in patients with cor pulmonale aged 40 years and above at high altitudes. This could help clinicians and especially general practitioners and residents to identify patients with poor prognosis at an early stage, and take the decision to address patients to hospital.

## Methods

### Study design and patients

Inpatients diagnosed with cor pulmonale at Qinghai Provincial People’s Hospital between January 1, 2012, and December 31, 2021, were retrospectively included. The study was approved by the Research Ethics Board at Qinghai Provincial People’s Hospital, Qinghai University (Ethical number: 2022-065). The requirement for individual informed consent was waived as this was a retrospective study.

**Fig. 1:**
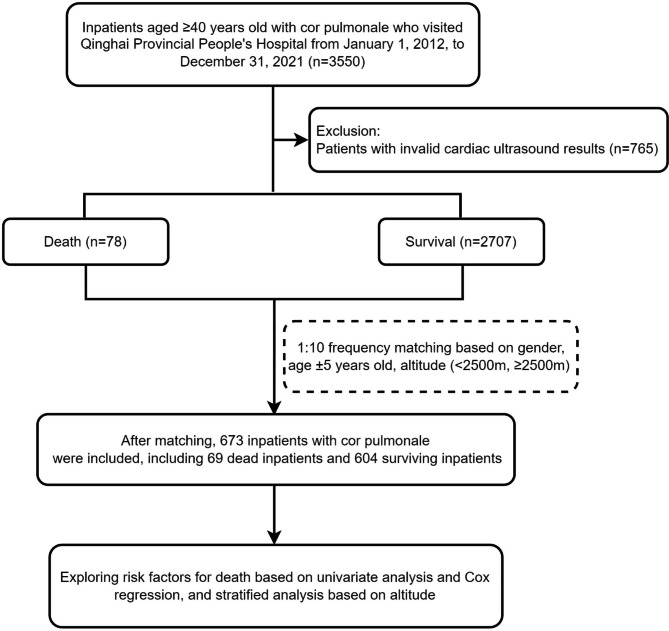
Flowchart of this study

The inclusion criteria were (1) diagnosed with cor pulmonale according to the Chinese Guideline for Primary Care of Chronic Cor Pulmonale [12] and (2) aged ≥ 40 years. Then, we excluded those did not have available cardiac ultrasound data. All participants were included as of December 31, 2021. Fifty-day mortality was chosen for a more comprehensive inclusion as patients with cor pulmonale tend to have longer lengths of stay [13]. And we had divided the above population into the death group the and survival group. To effectively explore the risk factors of death, we matched the death group and survival group with a frequency of 1:10 based on gender, age ± 5 years, and altitude group (< 2500 m, ≥ 2500 m). Finally, 673 patients with cor pulmonale were included in the analysis (Fig. 1).

### Data collection

The baseline characteristics of the patients were collected from the medical records, including age, sex, body mass index (BMI), smoking history, ethnicity, altitude of the living area, disease course, hospital stay, number of patients in acute exacerbation phase (respiratory symptoms and cardiac insufficiency worsened substantially than before), NYHA stage, previous history, treatment history, and complications. The symptoms, physical examination findings, laboratory examination findings, and treatments were collected.

The patients were categorized into survival and death groups according to their survival. The follow-up started from the diagnosis of cor pulmonale and ended at death, lost-to-follow-up, or August 31, 2022, whichever came first. According to the Qinghai criteria for high altitude sickness, subgroup analysis was performed using the cut-off altitude of 2500 m [14, 15],

### Statistical analysis

All statistical analyses were performed using SAS 9.4 (SAS Institute, Cary, NC, USA) and R (version 3.6.3, https://www.r-project.org/). The normality of the continuous variables was tested using the Kolmogorov-Smirnov test. The data in a normal distribution were described as means ± standard deviations and compared using the independent students’ *t*-test. The data not in a normal distribution were described as medians (ranges) and compared using the nonparametric Wilcoxon test. The categorical data were described as n (%) and compared using the chi-square test or Fisher exact probability test. We used the Kaplan-Meier curve to show the survival of different groups and log-rank tests were used to evaluate the difference. The survival of patients with cor pulmonale was used as the dependent variable for Cox regression analyses to explore the factors influencing the outcomes of the patients. The Schoenfeld residual was used to test whether the variable conformed to the assumption of proportional hazards. Candidate variables in multivariable analysis were selected based on the following criteria: (1) significantly related to death in univariate analysis; (2) clinical experience and the final model was determined according to the Akaike information criterion (AIC). Furthermore, we explored whether there was a multiplicative interaction between the altitude group (< 2500 m, ≥ 2500 m) and other independent variables. The interaction between the continuous variable (D-dimer) and altitude was visualized based on the parameter estimation of the Cox model. Based on the interaction we found, we further performed stratified analyses based on the altitude group. We used a two-tailed test, and P < 0.05 was considered statistically significant.

## Results

### Characteristics of the patients

The characteristics of the patients are shown in Tables 1, 2 and 3. Compared with the survival group, the death group showed a shorter length of stay, higher frequency of NYHA class IV, type II respiratory failure, pulmonary encephalopathy, and acid-base imbalance (*P* < 0.05) (Table S1). Moreover, a higher proportion of cardiac injury (68.2% vs. 39.6%, *P* < 0.001) and acute renal insufficiency (20.4% vs. 8.1%, *P* = 0.013) can be observed in the death group at an altitude of < 2500 m, but this phenomenon was not observed at an altitude of ≥ 2500 m.

**Table 1:** Sociodemographic and disease characteristics of patients with cor pulmonale

Variables	All (n = 673)	Altitude < 2500 m (n = 451)			Altitude ≥ 2500 m (n = 222)		
		Survival (n = 407)	Death (n = 44)	*P*	Survival (n = 197)	Death (n = 25)	*P*
Males	462 (68.6)	280 (68.8)	28 (63.6)	0.485	135 (68.5)	19 (76.0)	0.445
Age, years	73 ± 9	74 ± 8	76 ± 6	**0.033**	70 ± 11	69 ± 12	0.771
Han nationality	503 (74.7)	335 (82.3)	34 (77.3)	0.411	116 (58.9)	19 (72.0)	0.207
BMI, kg/m^2^	22.69 ± 4.61	23.01 ± 4.74	21.77 ± 5.09	0.104	22.32 ± 4.25	22.03 ± 4.11	0.748
Smoked	271 (40.3)	170 (41.8)	18 (40.9)	0.912	76 (38.6)	7 (28.0)	0.303
Admission within 10 years of onset	400 (59.4)	244 (59.9)	24 (54.5)	0.488	114 (57.9)	18 (72.0)	0.175
Length of stay, days	14 (11, 18)	14 (11, 19)	12 (6, 17)	**0.009**	13 (11, 17)	10 (6, 13)	**0.002**
Acute onset	346 (51.4)	219 (53.8)	24 (54.5)	0.926	86 (43.6)	17 (68.0)	**0.021**
NYHA class IV	153 (22.7)	75 (18.4)	23 (52.3)	**< 0.001**	42 (21.3)	13 (52.0)	**< 0.001**
**Disease history**							
COPD	293 (43.5)	192 (47.2)	18 (40.9)	0.429	72 (36.5)	11 (44.0)	0.468
Asthma	25 (3.7)	20 (4.9)	0 (0.0)	0.242	4 (2.0)	1 (4.0)	0.453
Tuberculosis	75 (11.4)	34 (8.3)	11 (25.0)	**0.002**	28 (14.2)	2 (8.0)	0.543
Interstitial lung disease	33 (4.9)	22 (5.4)	3 (6.8)	0.725	4 (2.0)	4 (16.0)	**0.006**
Thoracic or spinal deformity	64 (9.5)	39 (9.6)	3 (6.8)	0.785	14 (7.1)	8 (32.0)	**< 0.001**
Coronary heart disease	87 (12.9)	61 (15.0)	6 (13.6)	0.811	19 (9.6)	1 (4.0)	0.708
Diabetes	88 (13.1)	60 (14.7)	7 (15.9)	0.836	21 (10.7)	0 (0.0)	0.141
Hypertension	294 (43.7)	192 (47.2)	15 (34.1)	0.098	81 (41.1)	6 (24.0)	0.099
Pulmonary hypertension	105 (15.6)	70 (17.2)	2 (4.5)	**0.029**	27 (13.7)	6 (24.0)	0.227
**Treatment history**							
Theophylline	33 (4.9)	25 (6.1)	1 (2.3)	0.496	7 (3.5)	0 (0.0)	1.000
Diuretic	36 (5.3)	23 (5.6)	3 (6.8)	0.731	9 (4.6)	1 (4.0)	1.000
**Comorbidities**							
Edema of both lower extremities	394 (58.5)	241 (59.2)	29 (65.9)	0.389	109 (55.3)	15 (60.0)	0.658
Bronchitis	392 (58.2)	238 (58.5)	17 (38.6)	**0.012**	128 (65.0)	9 (36.0)	**0.005**
Emphysema	412 (61.2)	243 (59.7)	25 (56.8)	0.711	132 (67.0)	12 (48.0)	0.061
Bullae	181 (26.9)	111 (27.3)	12 (27.3)	1.000	51 (25.9)	7 (28.0)	0.821
Interstitial pneumonia	379 (56.3)	229 (56.3)	24 (54.5)	0.874	116 (58.9)	10 (40.0)	0.073
Pericardial effusion	120 (17.8)	60 (14.7)	9 (20.4)	0.317	44 (22.3)	7 (28.0)	0.526
Tricuspid regurgitation	604 (89.7)	407 (89.4)	43 (97.7)	0.105	176 (89.3)	21 (84.0)	0.497
Respiratory failure				**< 0.001**			**< 0.001**
Without	231 (34.2)	150 (36.9)	8 (18.2)		73 (37.1)	0 (0.0)	
Type I	301 (44.7)	180 (44.2)	16 (36.4)		91 (46.2)	14 (56.0)	
Type II	141 (20.9)	77 (18.9)	20 (45.4)		33 (16.7)	11 (44.0)	
Pulmonary encephalopathy	25 (3.7)	6 (1.5)	12 (27.3)	**< 0.001**	2 (1.0)	5 (20.0)	**< 0.001**
Pulmonary embolism	55 (8.2)	34 (8.3)	5 (11.4)	0.568	12 (6.1)	4 (16.0)	0.089
Cardiac injury	264 (39.2)	161 (39.6)	30 (68.2)	**< 0.001**	68 (34.5)	5 (20.0)	0.145
Acute renal insufficiency	59 (8.8)	33 (8.1)	9 (20.4)	**0.013**	13 (6.6)	4 (16.0)	0.108
Liver insufficiency	90 (13.4)	44 (10.8)	6 (13.6)	0.611	33 (16.7)	7 (28.0)	0.173
Electrolyte acid-base balance disorder	286 (42.5)	155 (38.1)	31 (70.4)	**< 0.001**	83 (42.1)	17 (68.0)	**0.014**

BMI: body mass index; NYHA: New York Heart Association Functional Classification; COPD: chronic obstructive pulmonary disease

**Table 2:** Symptoms and treatment of patients with cor pulmonale

Variables	All (n = 673)	Altitude < 2500 m (n = 451)			Altitude ≥ 2500 m (n = 222)		
		Survival (n = 407)	Death (n = 44)	*P*	Survival (n = 197)	Death (n = 25)	*P*
**Symptoms**							
Fever	82 (12.2)	50 (12.3)	3 (6.8)	0.285	20(10.1)	9 (36.0)	0.002
Cough	639 (94.9)	390 (95.8)	42 (95.4)	0.707	184 (93.4)	23 (92.0)	0.679
Expectoration	635 (94.3)	388 (95.3)	42 (95.4)	1.000	182 (92.4)	23 (92.0)	1.000
Wheeze	649 (96.4)	392 (96.3)	42 (95.4)	0.677	191 (96.9)	24 (96.0)	0.572
Dyspnea after exercise	406 (60.3)	240 (59.0)	32 (72.7)	0.076	121 (61.4)	13 (52.0)	0.364
Fatigue	360 (53.5)	217 (53.3)	32 (72.7)	**0.014**	107 (54.3)	4 (16.0)	**0.003**
Palpitations	138 (20.5)	92 (22.6)	4 (9.1)	**0.037**	41 (20.8)	1 (4.0)	0.055
Loss of appetite	322 (47.8)	190 (46.7)	15 (34.1)	0.111	101 (51.3)	16 (64.0)	0.230
Bloating	52 (7.7)	33 (8.1)	1 (2.3)	0.232	16 (8.1)	2 (8.0)	1.000
Nausea	30 (4.5)	21 (5.2)	2 (4.5)	1.000	5 (2.5)	2 (8.0)	0.180
**Treatment**							
High flux inhalation	79 (11.7)	46 (11.3)	16 (36.4)	**< 0.001**	11 (5.6)	6 (24.0)	**0.006**
Ventilation	88 (13.1)	44 (10.8)	17 (38.6)	**< 0.001**	16 (8.1)	11 (44.0)	**< 0.001**
Antibiotics	563 (83.7)	326 (80.1)	42 (95.4)	**0.012**	171 (86.8)	24 (96.0)	0.327
Antifungal drugs	83 (12.3)	44 (10.8)	16 (36.4)	**< 0.001**	18 (9.1)	5 (20.0)	0.152
Expectorant treatment	566 (84.1)	344 (84.5)	41 (93.2)	0.123	162 (82.2)	19 (76.0)	0.422
Methylprednisolone	103 (15.3)	59 (14.5)	14 (31.8)	**0.003**	24 (12.2)	6 (24.0)	0.120
Theophylline drugs	612 (90.9)	371 (91.1)	39 (88.6)	0.580	181 (91.9)	21 (84.0)	0.255
Diuretic therapy	431 (64.0)	248 (60.9)	36 (81.8)	**0.006**	127 (64.5)	20 (80.0)	0.122
Cardiotonic drugs	199 (29.6)	109 (26.8)	32 (72.7)	**< 0.001**	45 (22.8)	13 (52.0)	**0.002**
Vasodilation therapy	415 (61.7)	260 (63.9)	34 (77.3)	0.076	113 (57.4)	8 (32.0)	**0.016**
LMWH therapy	229 (34.0)	111 (27.3)	30 (68.2)	**< 0.001**	63 (32.0)	25 (100.00)	**< 0.001**
Inhalation medication	136 (20.2)	78 (19.2)	14 (31.8)	**0.048**	30 (15.2)	14 (56.0)	**< 0.001**

**Table 3:** Physical examination and laboratory test indexes in patients with cor pulmonale

Variables	All (n = 673)	Altitude < 2500 m (n = 451)			Altitude ≥ 2500 m (n = 222)		
		Survival (n = 407)	Death (n = 44)	*P*	Survival (n = 197)	Death (n = 25)	*P*
**Physical examination**							
Systolic blood pressure, mmHg	126 ± 21	126 ± 21	119 ± 20	0.058	129 ± 21	117 ± 23	**0.007**
Diastolic blood pressure, mmHg	77 ± 14	76 ± 14	74 ± 12	0.354	81 ± 13	73 ± 16	**0.010**
Heart rate, beats/min	87 ± 17	86 ± 15	95 ± 19	**< 0.001**	87 ± 19	93 ± 20	0.148
Respiratory rate, breaths/min	21 (20, 22)	21 (20, 22)	22 (20, 23)	**0.009**	20 (19, 22)	21 (20, 23)	0.175
**Laboratory test**							
PH	7.43 (7.39, 7.46)	7.43 (7.40, 7.46)	7.41 (7.34, 7.46)	0.091	7.43 (7.40, 7.46)	7.43 (7.36, 7.47)	0.959
PaO_2_, mmHg	59 (50, 72)	60 (50, 72)	56 (47, 75)	0.448	60 (50, 71)	56 (41, 70)	0.278
PaCO_2_, mmHg	40 (34, 47)	40 (34, 47)	42 (33, 57)	0.125	39 (35, 47)	40 (33, 53)	0.872
PaO_2_/FiO_2_	187 (152, 226)	189 (154, 229)	174 (140, 224)	0.126	185 (155, 223)	138 (116, 222)	**0.014**
White blood cell count, ×10^9^	5.89 (4.64, 7.55)	5.85 (4.69, 7.53)	6.46 (4.95, 10.09)	0.060	5.46 (4.50, 7.26)	6.08 (4.87, 9.83)	0.107
Neutrophil count, ×10^9^	4.15 (3.09, 5.93)	4.18 (3.13, 5.93)	5.20 (3.73, 8.26)	**0.009**	3.89 (2.85, 5.34)	4.15 (3.48, 7.10)	0.384
Eosinophil count, ×10^9^	0.06 (0.02, 0.14)	0.06 (0.03, 0.15)	0.04 (0.01, 0.12)	0.065	0.06 (0.02, 0.12)	0.09 (0.02, 0.15)	0.379
Hemoglobin, g/L	164 (142, 186)	163 (142, 184)	162 (135, 179)	0.248	170 (146, 195)	160 (126, 199)	0.318
Platelet count, ×10^9^	139 (99, 183)	146 (107, 189)	150 (97, 182)	0.638	124 (91, 169)	118 (83, 174)	0.926
Erythrocyte sedimentation rate, mm/h	4 (2, 14)	3 (2, 13)	11 (2, 20)	**0.012**	3 (1, 13)	13 (2, 17)	**0.032**
C-reactive protein, mg/L	1.64 (0.48, 4.92)	1.37 (0.44, 4.81)	3.80 (2.04, 5.81)	**0.002**	1.49 (0.43, 4.22)	5.56 (3.09, 8.28)	**< 0.001**
D-dimer, mg/L	1.64 (1.04, 3.02)	1.61 (1.03, 2.82)	3.14 (1.65, 4.87)	**< 0.001**	1.48 (0.96, 2.89)	3.08 (1.56, 4.07)	**0.001**
PT, s	13.3 (12.2, 14.8)	13.1 (12.2, 14.7)	14.4 (12.7, 17.5)	**0.003**	13.2 (12.2, 14.8)	14.5 (13.4, 16.0)	**0.002**
APTT, s	31.8 (27.7, 36.2)	31.1 (27.5, 35.6)	32.6 (28.6, 39.1)	0.058	32.0 (27.9, 36.7)	34.4 (30.7, 36.7)	0.258
Total protein, g/L	62.5 (58.0, 67.3)	62.8 (58.4, 67.8)	62.8 (55.8, 67.3)	0.434	61.1 (56.9, 66.7)	62.7 (59.3, 66.1)	0.405
Albumin, g/L	34 (31, 38)	35 (32, 38)	32 (28, 36)	**0.002**	34 (31, 37)	32 (30, 34)	0.068
ALT, U/L	19 (12, 37)	19 (12, 33)	28 (14, 74)	**0.033**	19 (13, 35)	29 (12, 69)	0.162
AST, U/L	25 (18, 40)	24 (18, 38)	30 (20, 101)	**0.003**	25 (19, 39)	34 (27, 59)	**0.016**
Uric acid, µmol/L	416 (313, 535)	421 (324, 538)	411 (301, 579)	0.961	395 (307, 520)	396 (293, 533)	0.849
Creatinine, µmol/L	77 (61, 98)	79 (61, 100)	95 (68, 121)	**0.027**	71 (61, 89)	73 (59,91)	0.938
LA diameter, mm	37 (33, 41)	37 (33, 41)	35 (31, 40)	0.056	37 (33, 41)	35 (31, 40)	0.491
LVESVI, mL/m^2^	44 (40, 48)	44 (40, 48)	42 (36, 45)	**0.005**	44 (40, 48)	44 (40, 49)	0.942
LVEDVI, mL/m^2^	27 (25, 31)	27 (25, 30)	26 (24, 30)	0.290	28 (25, 31)	27 (23, 31)	0.385
Simpson biplane EF, %	66 (61, 69)	66 (61, 70)	65 (60, 68)	0.097	66 (61, 70)	64 (60, 70)	0.779
RA diameter, mm	43 (38, 48)	42 (37, 47)	44 (41, 52)	**0.006**	43 (39, 48)	41 (40, 49)	0.950
RV diameter, mm	30 (26, 37)	30 (25, 36)	30 (26, 37)	0.510	32 (27, 37)	36 (30, 39)	**0.049**
RVWT, mm	5 (5, 6)	5 (5, 5)	5 (5, 6)	**0.017**	5 (5, 6)	5 (5, 6)	0.795
PA diameter, mm	28 (24, 31)	28 (24, 30)	29 (25, 31)	0.249	28 (24, 30)	30 (27, 33)	**0.021**
PASP, mmHg	60 (47, 75)	60 (46, 75)	64 (55, 77)	0.079	59 (47, 75)	66 (50, 74)	0.434

PaO_2_: partial pressure of oxygen; PaCO_2_: partial pressure of carbon dioxide; PaO_2_/FiO_2_: the ratio of arterial oxygen partial pressure to fractional inspired oxygen; PT: prothrombin time; APTT: activated partial thromboplastin time; ALT: alanine transaminase; AST: aspartate aminotransferase; LA: left atrial; LVESVI: left ventricular end-systolic volume index; LVEDVI: left ventricular end-diastolic volume index; EF: ejection fraction; RA: right atrial; RV: right ventricular; RVWT: right ventricular wall thickness; PA: pulmonary artery; PASP: pulmonary arterial systolic pressure

Regarding the symptoms, palpitations were more common in the survival group compared with the death group (22.0% vs. 7.2%, *P* = 0.004), especially at altitude < 2500 m (22.6% vs. 9.1%, *P* = 0.037). In addition, fatigue is more common among dead patients in areas with an altitude < 2500 m (*P* = 0.014). Compared with the survival group, the death group was treated more aggressively, with higher use of high flux inhalation, ventilation, antibiotics, antifungals, methylprednisolone, diuretics, cardiotonic drugs, inhalation medication, and low-molecular-weight heparin at altitude < 2500 m (all *P* < 0.05). And a higher proportion of using high flux inhalation, ventilation, cardiotonic drugs, vasodilation therapy, inhalation medication, and low-molecular-weight heparin was associated with death at an altitude ≥ 2500 m (Table 2 and Table S2).

Compared with the survival group, the death group showed higher erythrocyte sedimentation (*P* = 0.001), C-reactive protein (CRP) (*P* < 0.001), D-dimer levels (*P* < 0.001), prothrombin time (PT) (*P* < 0.001), and AST (*P* < 0.001) (Table S3). At altitude < 2500 m, the death group showed a lower systolic blood pressure (*P* = 0.007), diastolic blood pressure (*P* = 0.010), and PaO_2_/FiO_2_ (*P* = 0.014), and a higher RV diameter (*P* = 0.049) and PA diameter (*P* = 0.021) compared with the survival group. At altitude ≥ 2500 m, dead patients had a higher heart rate (*P* < 0.001), respiratory rate (*P* = 0.009), neutrophil count (*P* = 0.009), albumin (*P* = 0.002), ALT (*P* = 0.033), creatinine (*P* = 0.027) and LVESVI (*P* = 0.005) (Table 3).

### Overall survival

**Fig. 2:**
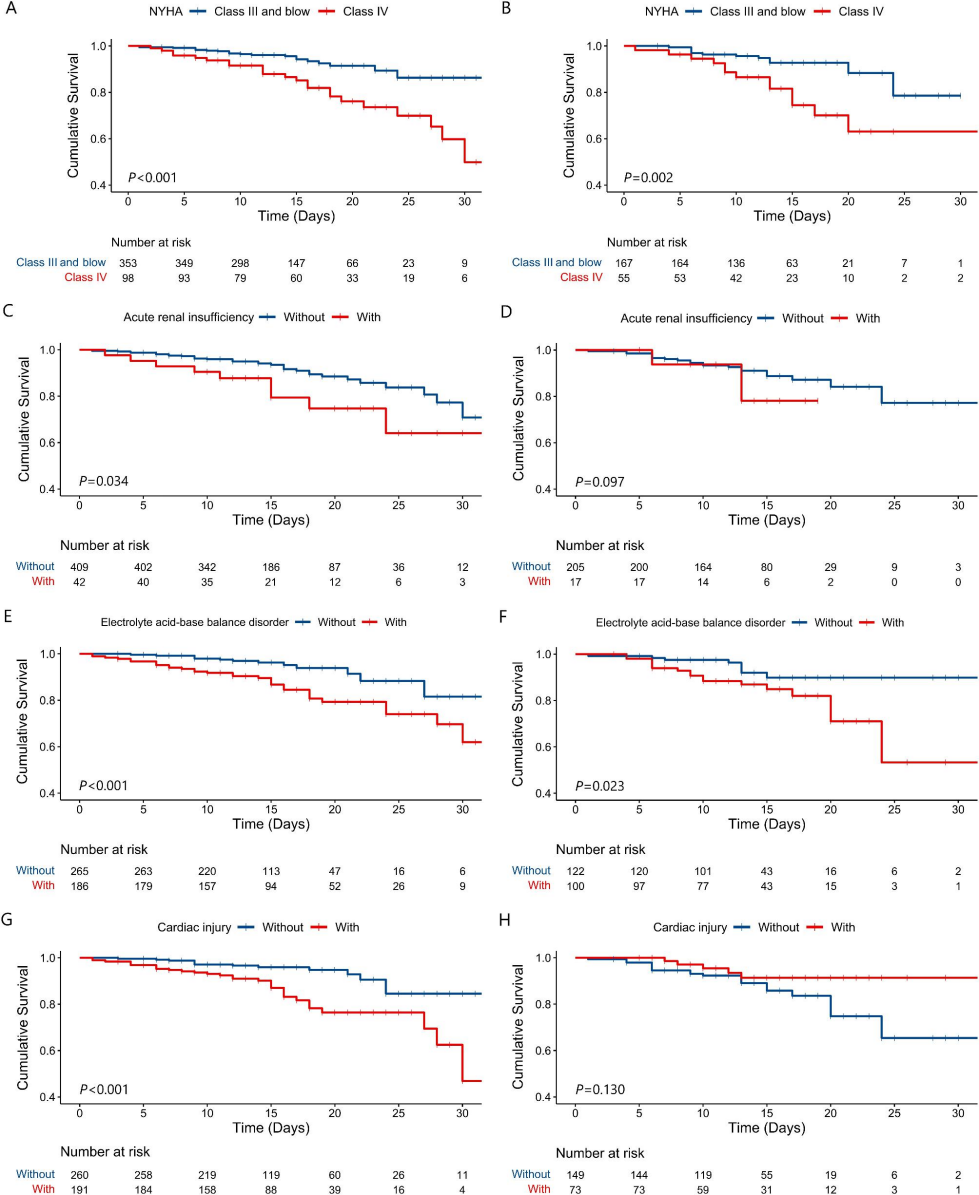
Kaplan-Meier curve of different subgroups at different altitudes. (**A**) New York Heart Association at altitude < 2500 m; (**B**) New York Heart Association at altitude ≥ 2500 m; (**C**) Acute renal insufficiency at altitude < 2500 m; (**D**) Acute renal insufficiency at altitude ≥ 2500 m; (**E**) Electrolyte acid-base balance disorder at altitude < 2500 m; (**F**) Electrolyte acid-base balance disorder at altitude ≥ 2500 m; (**G**) Cardiac injury at altitude < 2500 m; (**H**) Cardiac injury at altitude ≥ 2500 m

In all patients, the log-rank test showed those with NYHA class IV (*P* < 0.001), type II respiratory failure (*P* < 0.001), acute renal insufficiency (*P* = 0.008), acid-base imbalance (*P* < 0.001), and cardiac injury (*P* = 0.037) had worse survival (Figure S1). After altitude stratification, the association between acute renal insufficiency and heart injury and death was found only in people with an altitude of less than 2500 m (Fig. 2).

### Factors associated with death in cor pulmonale patients

The univariate Cox proportional hazards analysis showed that HYHA, respiratory failure, cardiac injury, acute renal insufficiency, electrolyte acid-base balance disorder, C-reactive protein, D-dimer, and PA diameter were associated with death. While at high altitudes, no association between heart injury and acute kidney injury and death was observed at high altitudes (Table 4).

**Table 4:** Univariate Cox proportional hazard analysis of death risk factors in patients with cor pulmonale

Variables	All patients (n = 673)		Altitude < 2500 m (n = 451)		Altitude ≥ 2500 m (n = 222)	
	HR (95% CI)	*P*	HR (95% CI)	*P*	HR (95% CI)	*P*
NYHA class IV	3.098 (1.923, 4.992)	< 0.001	3.018 (1.655, 5.503)	< 0.001	3.143 (1.432, 6.899)	0.004
Respiratory failure						
Type I	2.393 (1.095, 5.231)	0.029	-	-	-	-
Type II	4.772 (2.184, 10.428)	< 0.001	-	-	-	-
Cardiac injury	1.642 (1.024, 2.633)	0.040	3.184 (1.686, 6.013)	< 0.001	0.482 (0.181, 1.284)	0.144
Acute renal insufficiency	2.216 (1.211, 4.056)	0.010	2.170 (1.040, 4.526)	0.039	2.401 (0.820, 7.029)	0.110
Electrolyte acid-base balance disorder	2.765 (1.653, 4.625)	< 0.001	2.863 (1.492, 5.494)	0.002	2.548 (1.098, 5.915)	0.029
C-reactive protein, per mg/L	1.047 (1.012, 1.084)	0.008	1.044 (0.994, 1.096)	0.086	1.053 (1.003, 1.106)	0.036
D-dimer, per mg/L	1.101 (1.046, 1.158)	< 0.001	1.090 (1.027, 1.158)	0.005	1.173 (1.048, 1.313)	0.005
PA diameter, per mm	1.059 (1.015, 1.104)	0.007	1.052 (0.995, 1.112)	0.078	1.068 (1.005, 1.135)	0.033

- Indicates that the model with too few dead patients under stratification did not converge

**Fig. 3:**
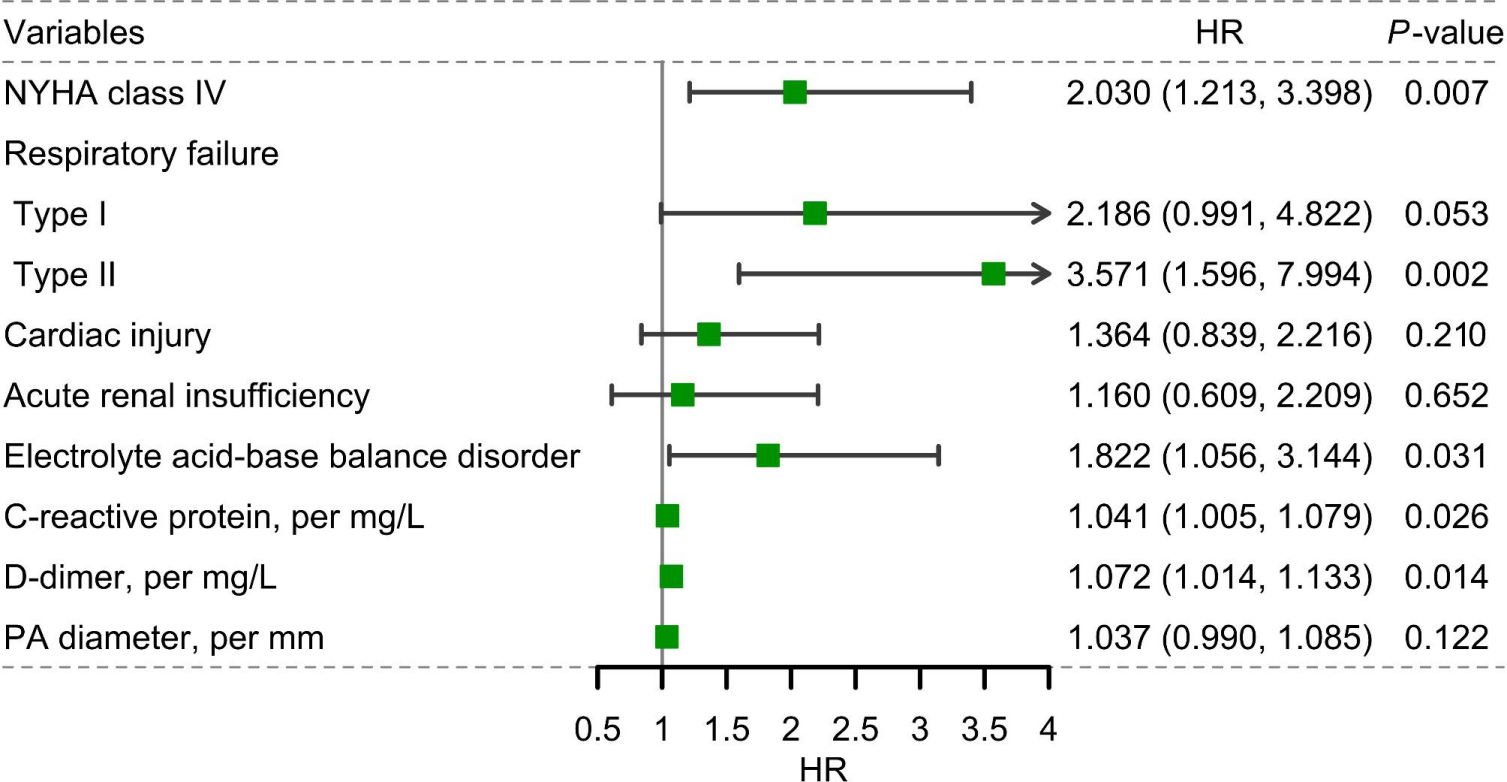
Forest plot for multivariate analysis of mortality risk of all participants

The multivariable Cox proportional hazards analysis showed that NYHA class IV (HR = 2.03, 95% CI: 1.21–3.40, *P* = 0.007), type II respiratory failure (HR = 3.57, 95%CI: 1.60–7.99, *P* = 0.002), acid-base imbalance (HR = 1.82, 95% CI: 1.06–3.14, *P* = 0.031), were risk factors for death in patients with cor pulmonale living at high altitude. The risk of death increases by 4% and 7% respectively for each unit of CRP (HR = 1.04, 95% CI: 1.01–1.08, *P* = 0.026) and D-dimer (HR = 1.07, 95% CI: 1.01–1.13, *P* = 0.014) increased (Fig. 3).

### Interaction of altitude with the risk factors

**Fig. 4:**
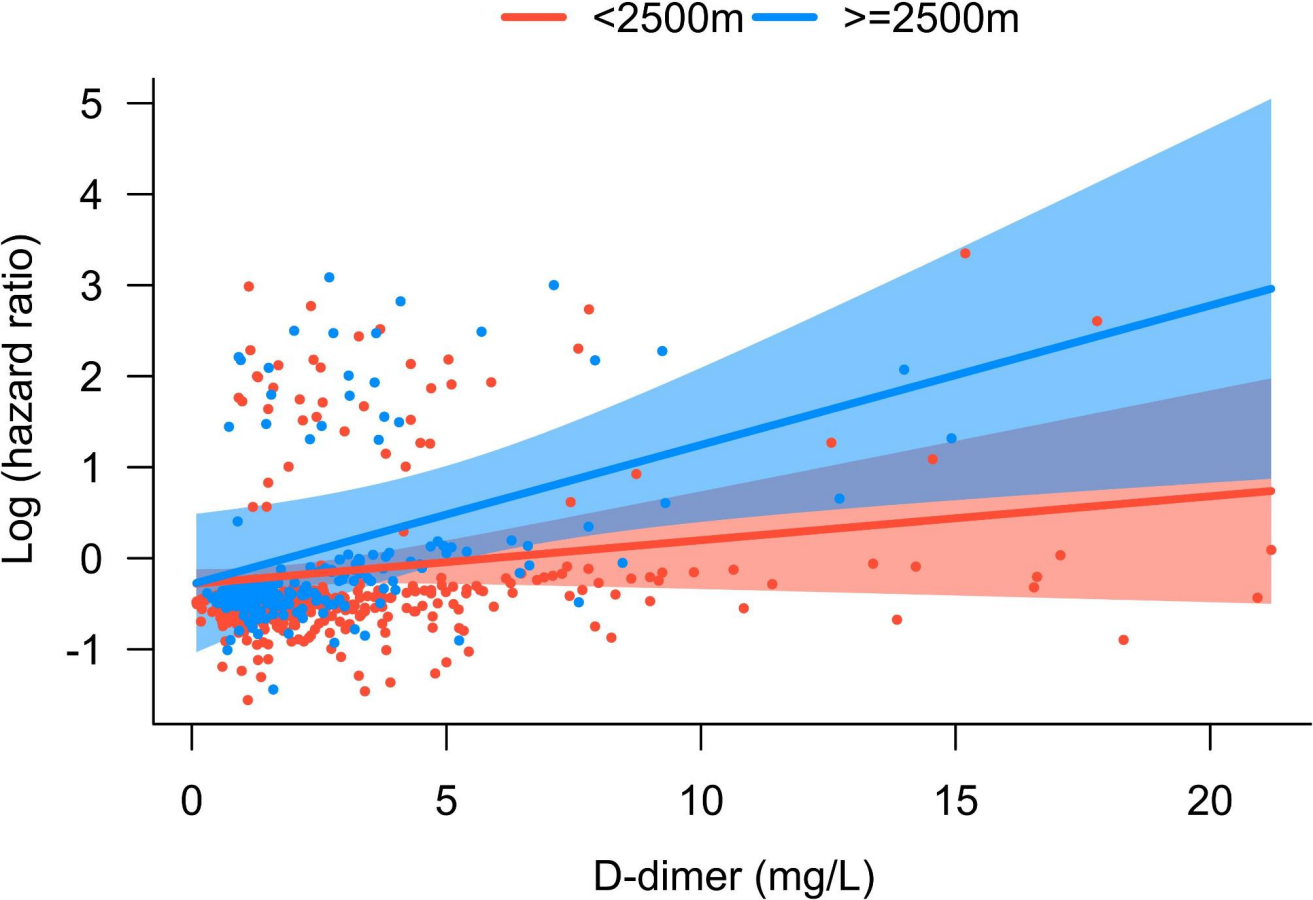
The interaction between D-dimer and death in patients with cor pulmonale at different altitudes

As shown in Table 5 and Figure S2, altitude interacted with cardiac injury (*P*_interaction_=0.006) and D-dimer levels (*P*_interaction_=0.031). Specifically, in patients living < 2500 m, cardiac injury was a risk factor for death (HR = 2.47, 95%CI: 1.28–4.77, *P* = 0.007), but it was not observed at > 2500 m (*P* = 0.057). For patients living at > 2500 m, every unit increase of D-dimer was associated with a 22.5% increase in mortality risk, but this association was not found in patients living at < 2500 m (Fig. 4).

**Table 5:** Multivariable Cox proportional hazard analysis of death risk factors of death in patients with cor pulmonale based on altitude stratification

Variables	Altitude < 2500 m (n = 451)		Altitude ≥ 2500 m (n = 222)		*P* for interaction
	HR (95% CI)	*P*	HR (95% CI)	*P*	
NYHA class IV	1.978 (1.054, 3.712)	**0.034**	2.900 (1.186, 7.091)	**0.020**	0.211
Cardiac injury	2.469 (1.279, 4.766)	**0.007**	0.363 (0.128, 1.030)	0.057	**0.006**
Acute renal insufficiency	1.133 (0.526, 2.442)	0.750	1.214 (0.383, 3.852)	0.742	0.865
Electrolyte acid-base balance disorder	2.212 (1.121, 4.364)	**0.022**	1.848 (0.770, 4.437)	0.169	0.965
C-reactive protein, per mg/L	1.026 (0.976, 1.079)	0.309	1.057 (1.004, 1.113)	**0.034**	0.438
D-dimer, per mg/L	1.050 (0.986, 1.118)	0.129	1.225 (1.069, 1.404)	**0.003**	**0.031**
PA diameter, per mm	1.027 (0.966, 1.092)	0.389	1.041 (0.965, 1.123)	0.302	0.153

HR: hazard ratio; CI: confidence interval; NYHA: New York Heart Association functional classification; PA: pulmonary artery

## Discussion

This study explored the risk factors mortality in patients with acute exacerbation of chronic cor pulmonale in plateau regions. The results suggested that NYHA class IV, type II respiratory failure, acid-base imbalance, CRP, and D-dimer were associated with death in patients with cor pulmonale. And the relationships between D-dimer, heart injury, and mortality were modified by altitude.

The prognosis and risk factors for death vary widely because of the various etiologies of cor pulmonary [16]. In a study of patients with chronic obstructive pulmonary disease and pulmonary hypertension, only the tricuspid annular plane systolic excursion/pulmonary artery systolic pressure (TAPSE/PASP) ratio and the 6-minute walking distance (6MWD) independently predicted mortality [17]. In patients with chronic obstructive pulmonary disease [18] or chronic lung diseases [19], the lung diffusing capacity for carbon monoxide (DLCO) is an important predictor of mortality in patients with pulmonary hypertension. Right heart failure is a complication of cor pulmonale. Sztrymf et al. [20] reported that elevated BNP, CRP, serum creatinine, SAPS II, and the presence were independently associated with increased mortality in patients presenting to the ICU with acute right heart failure. Haddad et al. [21] showed that lower serum sodium, elevated respiratory rate, low GFR, and worse tricuspid regurgitation severity were each associated with increased mortality in patients with pulmonary artery hypertension hospitalized for acute right heart failure.

Living at high altitudes induces several metabolic changes due to long-term chronic hypoxia [22]. Exposure to high altitude is associated with an increased metabolism and leptin levels [23]. Physiological changes include hyperventilation, increased heart rate, and increased red blood cell mass [24]. Since these factors are also contributed to pulmonary hypertension and cor pulmonale at high altitudes [25, 26], the risk factors for mortality might differ between people living on the plains and plateaus. At present, the relationship between altitude and the prognosis of patients with cor pulmonale is not clear, but a previous study suggested that chronic hypoxia at high altitude may be related to right ventricular hypertrophy and heart failure [27]. In this study, NYHA class IV, type II respiratory failure, acid-base imbalance, CRP, and D-dimer were risk factors for death in patients with cor pulmonale living at high altitudes. These factors are all associated with a higher mortality risk to various degrees in a large number of diseases [28–30].

Additionally, we found that type II respiratory failure was associated with an increased risk of death in patients with acute exacerbation of cor pulmonale (HR = 4.772, 95% CI: 2.184–10.428, P < 0.001). Some studies have shown that acute exacerbation of cor pulmonale patients with hypercapnia and respiratory acidosis often have insufficient alveolar ventilation due to injury to the diaphragm [31]. These patients have a higher intubation and mortality rate [32]. Therefore, the pulse oxymetry should be monitored strictly for the patients with cor pulmonale living at high altitudes.

Interestingly, the association between D-dimer and mortality in patients were only found in those living at > 2500 m (22.5% for each one-unit increase). Elevated D-dimer is associated with increased mortality in the general population [33], however, insufficient evidence in patients with cor pulmonale. D-dimer is produced when a blood clot dissolves, indicating a thrombosis event [34]. High altitude is associated with a higher risk of pulmonary embolism, cerebral venous thrombosis, portal/splenic vein thrombosis, and deep vein thrombosis, all of which are associated with increased mortality risk [35]. Massive pulmonary embolism is the most common cause of acute cor pulmonale [1–3]. Therefore, D-dimer levels should be evaluated in the clinical workup of patients with cor pulmonale. In addition, studies pointed out that the high-altitude environment had a significant impact on the fibrinolytic system [36], and the changes in the fibrinolytic system were associated with an increased risk of death [37].

This study had limitations. All included patients had cor pulmonale, and the factors associated with cor pulmonale could not be explored. It was a retrospective study limited to the data available in the charts. The occurrence of the event was dependent upon being documented in the charts, and there is a possibility of a patient dying at another hospital. In addition, because we matched age and sex, this may lead to selection bias, that is, the conclusion needs to be further verified in other populations.

## Conclusions

In conclusion, NYHA class IV, type II respiratory failure, acid-base imbalance, CRP, and D-dimer were risk factors for death in patients with cor pulmonale living at high altitudes. These results could help identify patients with cor pulmonale who might require more aggressive management.

## Electronic supplementary material

Below is the link to the electronic supplementary material.

Supplementary Material 1

Supplementary Material 2

Supplementary Material 3

## Data Availability

The anonymous dataset is available from the corresponding author.

## Abbreviations

ALT: Alanine transaminase
APTT: Activated partial thromboplastin time
AST: Aspartate aminotransferase
BMI: Body mass index
CI: Confidence interval
COPD: Chronic obstructive pulmonary disease
EF: Ejection fraction
HR: Hazard ratio
LA: Left atrial
LVEDVI: Left ventricular end-diastolic volume index
LVESVI: Left ventricular end-systolic volume index
NYHA: New York Heart Association Functional Classification
PA: Pulmonary artery
PA: Pulmonary artery
PaCO2: Partial pressure of carbon dioxide
PaO2/FiO2: The ratio of arterial oxygen partial pressure to fractional inspired oxygen
PaO2: Partial pressure of oxygen
PASP: Pulmonary arterial systolic pressure
PT: Prothrombin time
RA: Right atrial
RV: Right ventricular
RVWT: Right ventricular wall thickness
