# Supplementary material for: Risk factors for mortality in patients with acute exacerbation of cor pulmonale in plateau
Source: BMC Pulm Med. 2023 Jul 3;23:238. doi: 10.1186/s12890-023-02509-1 (PMC10318768; doi:10.1186/s12890-023-02509-1)
Supplement: Supplementary file 1 — Supplementary Material 1 [file 12890_2023_2509_MOESM1_ESM.docx]

**Table S1.** Sociodemographic and disease characteristics of patients with cor pulmonale

| Variables | All (n=673) | Survival (n=604) | Death (n=69) | *P* |
| --- | --- | --- | --- | --- |
| Males | 462 (68.6) | 415 (68.7) | 47 (68.1) | 0.920 |
| Age, years | 73±9 | 73±9 | 74±9 | 0.398 |
| Han nationality | 503 (74.7) | 451 (74.7) | 52 (75.4) | 0.900 |
| Altitude <2500 m | 451 (67.0) | 407 (67.4) | 44 (63.8) | 0.545 |
| BMI, kg/m^2^ | 22.69±4.61 | 22.78±4.59 | 21.87±4.73 | 0.118 |
| Smoked | 271 (40.3) | 246 (40.7) | 25 (36.2) | 0.471 |
| Admission within 10 years of onset | 400 (59.4) | 358 (59.3) | 42 (60.9) | 0.798 |
| Length of stay, days | 14 (11, 18) | 14 (11, 18) | 11 (6, 16) | <0.001 |
| Acute onset | 346 (51.4) | 305 (50.5) | 41 (59.4) | 0.160 |
| NYHA class IV | 153 (22.7) | 117 (19.4) | 36 (52.2) | <0.001 |
| Disease history |  |  |  |  |
| COPD | 293 (43.5) | 264 (43.7) | 29 (42.0) | 0.790 |
| Asthma | 25 (3.7) | 24 (4.0) | 1 (1.4) | 0.501 |
| Tuberculosis | 75 (11.4) | 62 (10.3) | 13 (18.8) | 0.032 |
| Interstitial lung disease | 33 (4.9) | 26 (4.3) | 7 (10.1) | 0.068 |
| Thoracic or spinal deformity | 64 (9.5) | 53 (8.8) | 11 (15.9) | 0.054 |
| Coronary heart disease | 87 (12.9) | 80 (13.2) | 7 (10.1) | 0.467 |
| Diabetes | 88 (13.1) | 81 (13.4) | 7 (10.1) | 0.446 |
| Hypertension | 294 (43.7) | 273 (45.2) | 21 (30.4) | 0.019 |
| Pulmonary hypertension | 105 (15.6) | 97 (16.1) | 8 (11.6) | 0.333 |
| Treatment history |  |  |  |  |
| Theophylline | 33 (4.9) | 32 (5.3) | 1 (1.4) | 0.239 |
| Diuretic | 36 (5.3) | 32 (5.3) | 4 (5.8) | 0.779 |
| Comorbidities |  |  |  |  |
| Edema of both lower extremities | 394 (58.5) | 350 (57.9) | 44 (63.8) | 0.352 |
| Bronchitis | 392 (58.2) | 366 (60.6) | 26 (37.7) | <0.001 |
| Emphysema | 412 (61.2) | 375 (62.1) | 37 (53.6) | 0.172 |
| Bullae | 181 (26.9) | 162 (26.8) | 19 (27.5) | 0.899 |
| Interstitial pneumonia | 379 (56.3) | 345 (57.1) | 34 (49.3) | 0.213 |
| Pericardial effusion | 120 (17.8) | 104 (17.2) | 16 (23.2) | 0.220 |
| Tricuspid regurgitation | 604 (89.7) | 540 (89.4) | 64 (92.7) | 0.385 |
| Respiratory failure |  |  |  | <0.001 |
| Without | 231 (34.2) | 223 (36.9) | 8 (11.6) |  |
| Type I | 301 (44.7) | 271 (44.9) | 30 (43.5) |  |
| Type II | 141 (20.9) | 110 (18.2) | 31 (44.9) |  |
| Pulmonary encephalopathy | 25 (3.7) | 8 (1.3) | 17 (24.6) | <0.001 |
| Pulmonary embolism | 55 (8.2) | 46 (7.6) | 9 (13.0) | 0.119 |
| Cardiac injury | 264 (39.2) | 229 (37.9) | 35 (50.7) | 0.039 |
| Acute renal insufficiency | 59 (8.8) | 46 (7.6) | 13 (18.8) | 0.002 |
| Liver insufficiency | 90 (13.4) | 77 (12.7) | 13 (18.8) | 0.160 |
| Electrolyte acid-base balance disorder | 286 (42.5) | 238 (39.4) | 48 (69.6) | <0.001 |

BMI: body mass index; NYHA: New York Heart Association Functional Classification; COPD: chronic obstructive pulmonary disease.

**Table S2.** Symptoms and treatment of all patients with cor pulmonale

| Variables | All (n=673) | Survival (n=604) | Death (n=69) | *P* |
| --- | --- | --- | --- | --- |
| Symptoms |  |  |  |  |
| Fever | 82 (12.2) | 70 (11.6) | 12 (17.4) | 0.163 |
| Cough | 639 (94.9) | 574 (95.0) | 65 (94.2) | 0.770 |
| Expectoration | 635 (94.3) | 570 (94.4) | 65 (94.2) | >0.999 |
| Wheeze | 649 (96.4) | 583 (96.5) | 66 (95.6) | 0.728 |
| Dyspnea after exercise | 406 (60.3) | 361 (59.8) | 45 (65.2) | 0.381 |
| Fatigue | 360 (53.5) | 324 (53.6) | 36 (52.2) | 0.817 |
| Palpitations | 138 (20.5) | 133 (22.0) | 5 (7.2) | 0.004 |
| Loss of appetite | 322 (47.8) | 291 (48.2) | 31 (44.9) | 0.608 |
| Bloating | 52 (7.7) | 49 (8.1) | 3 (4.3) | 0.267 |
| Nausea | 30 (4.5) | 26 (4.3) | 4 (5.8) | 0.536 |
| Treatment |  |  |  |  |
| High flux inhalation | 79 (11.7) | 57 (9.4) | 22 (31.8) | <0.001 |
| Ventilation | 88 (13.1) | 60 (9.9) | 28 (40.6) | <0.001 |
| Antibiotics | 563 (83.7) | 497 (82.3) | 66 (95.6) | 0.004 |
| Antifungal drugs | 83 (12.3) | 62 (10.3) | 21 (30.4) | <0.001 |
| Expectorant treatment | 566 (84.1) | 506 (83.8) | 60 (87.0) | 0.493 |
| Methylprednisolone | 103 (15.3) | 83 (13.7) | 20 (29.0) | <0.001 |
| Theophylline drugs | 612 (90.9) | 552 (91.4) | 60 (87.0) | 0.224 |
| Diuretic therapy | 431 (64.0) | 375 (62.1) | 56 (81.2) | 0.002 |
| Cardiotonic drugs | 199 (29.6) | 154 (25.5) | 45 (65.2) | <0.001 |
| Vasodilation therapy | 415 (61.7) | 373 (61.7) | 42 (60.9) | 0.886 |
| LMWH therapy | 229 (34.0) | 174 (28.8) | 55 (79.7) | <0.001 |

**Table S3.** Physical examination and laboratory test indexes in all patients with cor pulmonale

| Variables | All (n=673) | Survival (n=604) | Death (n=69) | *P* |
| --- | --- | --- | --- | --- |
| Physical examination |  |  |  |  |
| Systolic blood pressure, mmHg | 126±21 | 127±21 | 118±21 | 0.002 |
| Diastolic blood pressure, mmHg | 77±14 | 78±14 | 74±14 | 0.029 |
| Heart rate, beats/min | 87±17 | 86±16 | 95±19 | <0.001 |
| Respiratory rate, breaths/min | 21 (20, 22) | 20 (19, 22) | 21 (20, 23) | 0.004 |
| Laboratory test |  |  |  |  |
| PH | 7.43 (7.39, 7.46) | 7.43 (7.40, 7.46) | 7.43 (7.36, 7.47) | 0.166 |
| PaO_2_, mmHg | 59 (50, 72) | 60 (50, 72) | 56 (44, 75) | 0.192 |
| PaCO_2_, mmHg | 40 (34, 47) | 40 (34, 47) | 41 (33, 55) | 0.201 |
| PaO_2_/FiO_2_ | 187 (152, 226) | 187 (155, 226) | 161 (132, 223) | 0.007 |
| White blood cell count, ×10^9^ | 5.89 (4.64, 7.55) | 5.81 (4.61, 7.45) | 6.38 (4.94, 9.83) | 0.015 |
| Neutrophil count, ×10^9^ | 4.15 (3.09, 5.93) | 4.09 (3.06, 5.79) | 4.59 (3.56, 7.65) | 0.011 |
| Eosinophil count, ×10^9^ | 0.06 (0.02, 0.14) | 0.06 (0.02, 0.14) | 0.05 (0.01, 0.14) | 0.313 |
| Hemoglobin, g/L | 164 (142, 186) | 164 (142, 186) | 162 (135, 181) | 0.122 |
| Platelet count, ×10^9^ | 139 (99, 183) | 139 (100, 183) | 149 (94, 180) | 0.712 |

| Erythrocyte sedimentation rate, mm/h | 4 (2, 14) | 3 (1, 13) | 13 (2, 19) | 0.001 |
| --- | --- | --- | --- | --- |
| C-reactive protein, mg/L | 1.64 (0.48, 4.92) | 1.45 (0.43, 4.70) | 4.13 (2.33, 6.61) | <0.001 |
| D-dimer, mg/L | 1.64 (1.04, 3.02) | 1.57 (1.00, 2.83) | 3.08 (1.60, 4.08) | <0.001 |
| PT, s | 13.3 (12.2, 14.8) | 13.1 (12.2, 14.7) | 14.5 (13.2, 16.7) | <0.001 |
| APTT, s | 31.8 (27.7, 36.2) | 31.3 (27.5, 35.9) | 33.0 (29.1, 38.0) | 0.023 |
| Total protein, g/L | 62.5 (58.0, 67.3) | 62.4 (57.9, 67.4) | 62.7 (58.5, 66.7) | 0.832 |
| Albumin, g/L | 34 (31, 38) | 34 (31, 38) | 32 (29, 36) | <0.001 |
| ALT, U/L | 19 (12, 37) | 19 (12, 34) | 28 (14, 69) | 0.011 |
| AST, U/L | 25 (18, 40) | 24 (18, 38) | 31 (22, 97) | <0.001 |
| Uric acid, μmol/L | 416 (313, 535) | 416 (315, 534) | 402 (301, 553) | 0.839 |
| Creatinine, μmol/L | 77 (61, 98) | 76 (61, 95) | 83 (62, 115) | 0.098 |
| LA diameter, mm | 37 (33, 41) | 37 (33, 41) | 35 (31, 40) | 0.051 |
| LVESVI, mL/m^2^ | 44 (40, 48) | 44 (40, 48) | 43 (38, 47) | 0.024 |
| LVEDVI, mL/m^2^ | 27 (25, 31) | 27 (25, 31) | 27 (23, 30) | 0.180 |
| Simpson biplane EF, % | 66 (61, 69) | 66 (61, 70) | 65 (60, 68) | 0.137 |
| RA diameter, mm | 43 (38, 48) | 42 (38, 48) | 44 (40, 51) | 0.025 |
| RV diameter, mm | 30 (26, 37) | 30 (26, 36) | 31 (27, 37) | 0.078 |
| RVWT, mm | 5 (5, 6) | 5 (5, 5) | 5 (5, 6) | 0.082 |
| PA diameter, mm | 28 (24, 31) | 28 (24, 30) | 29 (26, 32) | 0.020 |
| PASP, mmHg | 60 (47, 75) | 60 (46, 75) | 65 (53, 75) | 0.063 |

PaO_2_: partial pressure of oxygen; PaCO_2_: partial pressure of carbon dioxide; PaO_2_/FiO_2_: the ratio of arterial oxygen partial pressure to fractional inspired oxygen; PT: prothrombin time; APTT: activated partial thromboplastin time; ALT: alanine transaminase; AST: aspartate aminotransferase; LA: left atrial; LVESVI: left ventricular end-systolic volume index; LVEDVI: left ventricular end-diastolic volume index; EF: ejection fraction; RA: right atrial; RV: right ventricular; RVWT: right ventricular wall thickness; PA: pulmonary artery; PASP: pulmonary arterial systolic pressure.

**Figure S1.** Kaplan-Meier curve of different subgroups in all patients. (A) New York Heart Association (NYHA). (B) Respiratory failure. (C) Acute renal insufficiency. (D) Electrolyte acid-base balance disorder. (E) Cardiac injury

**Figure S2.** Forest plot of the multivariable analysis of mortality risk in different altitude subgroups
