# Supplementary figures and images for: Risk factors for mortality in patients with acute exacerbation of cor pulmonale in plateau
Source: BMC Pulm Med. 2023 Jul 3;23:238. doi: 10.1186/s12890-023-02509-1 (PMC10318768; doi:10.1186/s12890-023-02509-1)

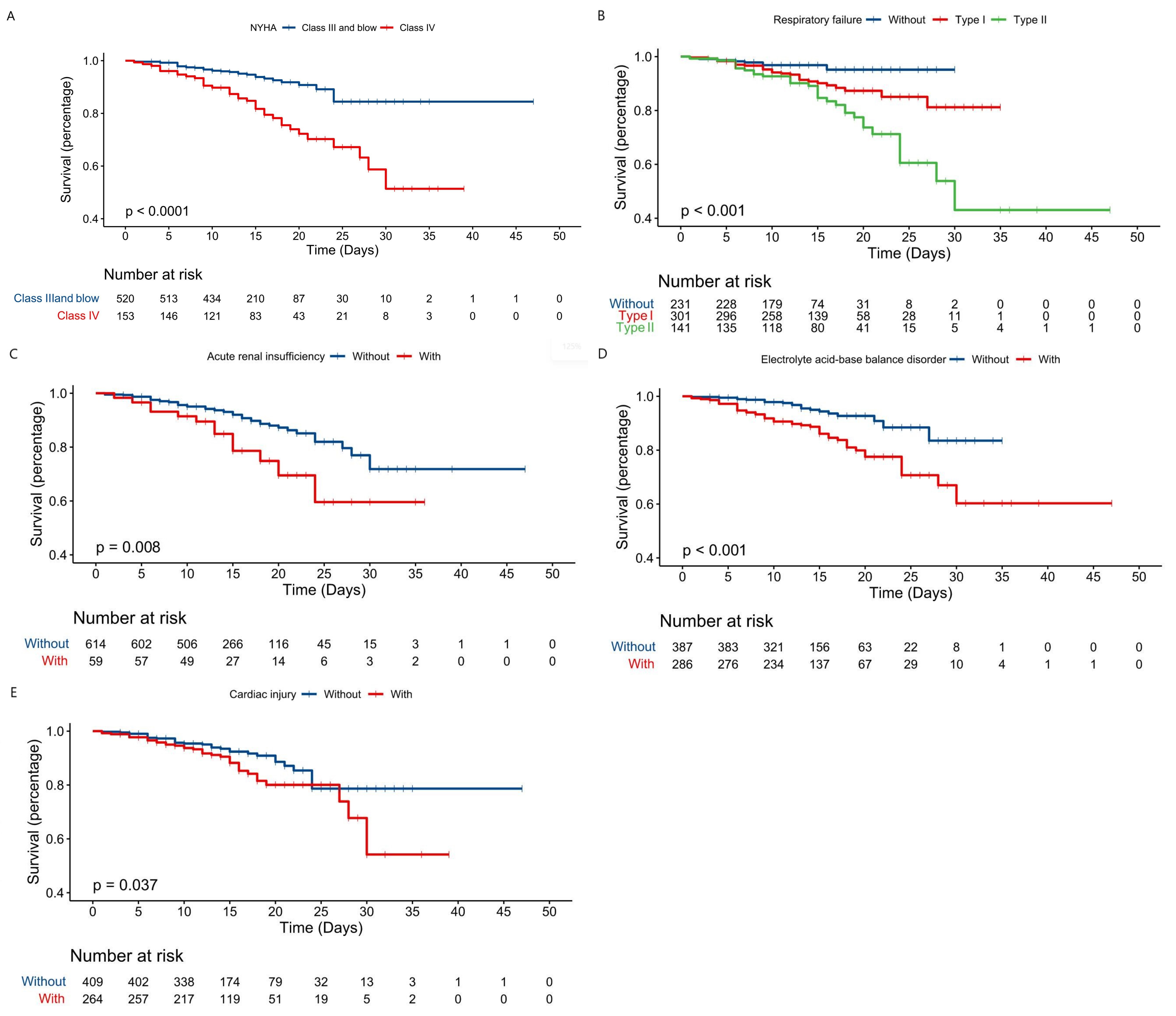

Supplement: Supplementary file 2 — Supplementary Material 2 [file 12890_2023_2509_MOESM2_ESM.jpg]

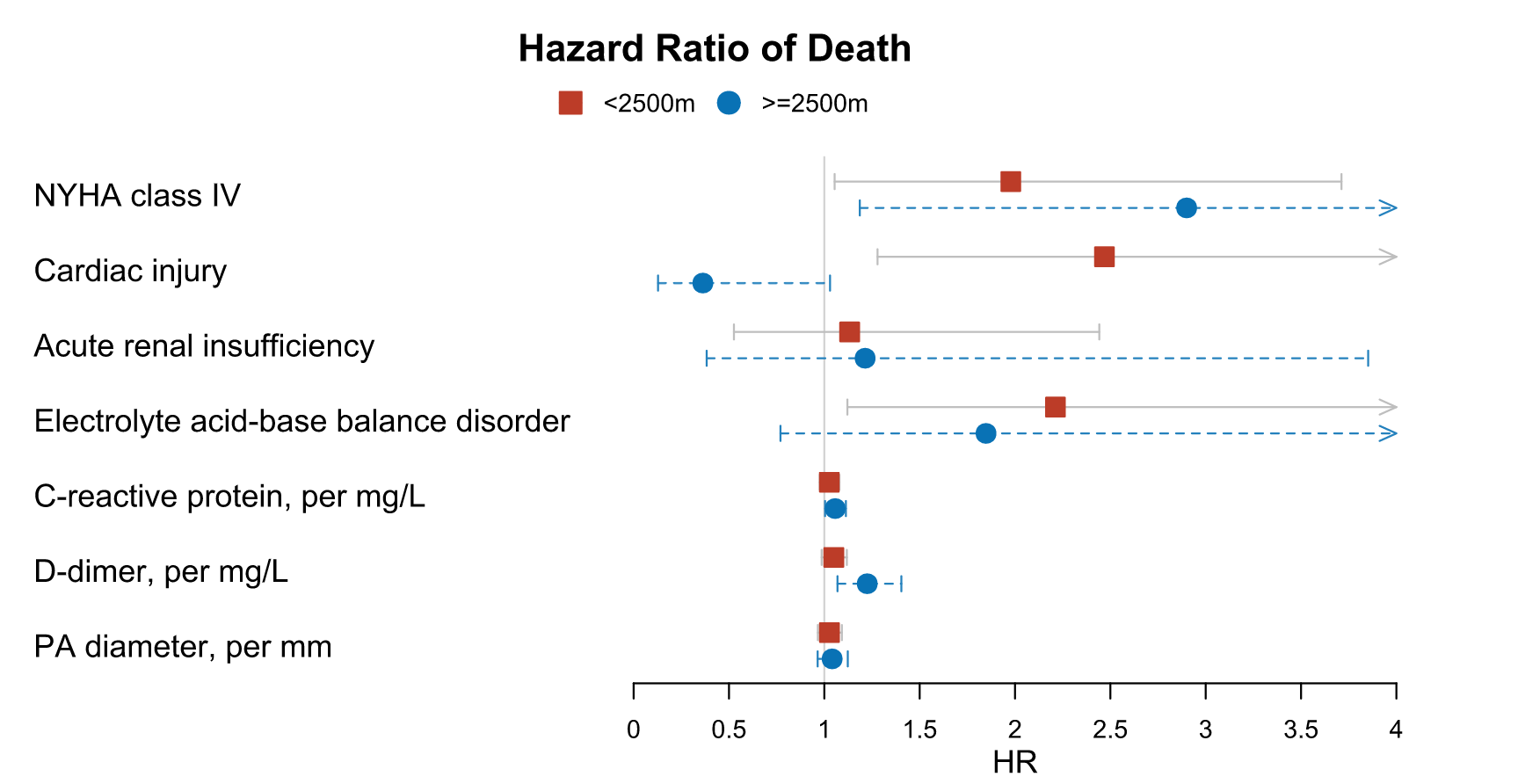

Supplement: Supplementary file 3 — Supplementary Material 3 [file 12890_2023_2509_MOESM3_ESM.png]
